# Impact of a Multifaceted Antimicrobial Stewardship Program on Antimicrobial Use and Prescription Quality in a Pediatric Intensive Care Unit: An Interrupted Time-Series Study

**DOI:** 10.3390/antibiotics15090907

**Published:** 2026-09-15

**Authors:** Laura Fernández-Vallespin, Elena Fresán-Ruiz, Maria Goretti López-Ramos, Ángela Pieras-López, Eneritz Velasco-Arnaiz, Iolanda Jordan

**Affiliations:** 1 Pediatric Intensive Care Unit, Hospital Sant Joan de Déu, Passeig Sant Joan de Déu, 2, 08950 Barcelona, Spain; laura.fernandezv@sjd.es (L.F.-V.); iolanda.jordan@sjd.es (I.J.); 2 Immunological and Respiratory Disorders in the Pediatric Critical Patient Research Group, Institut de Recerca Sant Joan de Déu, C. de Sta. Rosa, 39, 08950 Barcelona, Spain; 3 Pharmacy Department, Hospital Sant Joan de Déu, Passeig Sant Joan de Déu, 2, 08950 Barcelona, Spain; mariagoretti.lopez@sjd.es (M.G.L.-R.); angela.pieras@sjd.es (Á.P.-L.); 4 Infectious Diseases and Systemic Inflammatory Response in Pediatrics, Pediatric Infectious Diseases Department, Institut de Recerca Sant Joan de Déu, Hospital Sant Joan de Déu, Passeig Sant Joan de Déu, 2, 08950 Barcelona, Spain; eneritz.velasco@sjd.es; 5 Pediatrics Department, University of Barcelona (UB), C. de Casanova, 143, 08036 Barcelona, Spain; 6 Consorcio de Investigación Biomédica en Red de Epidemiología y Salud (CIBER), C. de Melchor Fernández Almagro, 3, Fuencarral-El Pardo, 28029 Madrid, Spain

**Keywords:** antimicrobial stewardship, intensive care units, pediatric, antibiotics, antibiotic

## Abstract

Background: Inappropriate antibiotic use contributes to antimicrobial resistance. Evidence regarding the impact of antimicrobial stewardship programs (ASPs) in pediatric intensive care units (PICUs) remains limited. The study aims to evaluate the impact of a multidisciplinary prospective post-prescription review and feedback (PPRF) ASP in a European PICU on antimicrobial use and prescription quality (PQ). Methods: A prospective quasi-experimental study was conducted using interrupted time-series analysis to evaluate antimicrobial use trends before (July 2018–December 2020) and after (January 2021–March 2025) the implementation of a multifaceted ASP, integrated within a hospital-wide ASP, in a tertiary 24-bed medical–surgical PICU in Barcelona, Spain. Antimicrobial use was measured as days of therapy per 100 patient-days (DOT/100 PD) and per 100 discharges (DOT/100 D) and was analyzed by WHO AWaRe group and by individual drug. PQ was evaluated by means of cross-sectional point-prevalence surveys (PPSs). Results: During the study-period, median monthly activity in the PICU remained stable: 475 patient-days (IQR: 396–534) and 113 patients discharged (IQR: 102–124). Following ASP implementation, total antibiotic consumption significantly decreased, with an immediate reduction of 18.2 DOT/100 patient-days (*p* = 0.006) and a sustained monthly decline of 0.73 DOT/100 patient-days (*p* = 0.028). The COVID-19 pandemic did not significantly affect overall antibiotic consumption. *Access* and *Reserve* antibiotic use decreased significantly after ASP implementation, whereas *Watch* antibiotic use remained unchanged. Overall antifungal consumption was not significantly modified, although a transient increase was observed during the pandemic. At the individual drug level, several antibiotics demonstrated significant immediate decreases after ASP implementation. Prescription quality remained high (>88% optimal prescriptions), and PICU length of stay and mortality were unchanged. Conclusions: Implementation of a multidisciplinary PPRF-based ASP in a tertiary PICU was associated with a sustained reduction in antibiotic exposure without compromising prescription quality or clinical safety. These data support the effectiveness of collaborative stewardship in one of the most complex pediatric healthcare settings.

## 1. Introduction

Antibiotic therapy has substantially reduced infection-related morbidity and mortality in critically ill children [1]. However, its widespread and empirical use persists in pediatric intensive care units (PICUs), as the overlap between infectious and non-infectious inflammatory conditions often delays or precludes definitive confirmation of infection. This diagnostic uncertainty frequently leads to antimicrobial overtreatment, contributing to the emergence and spread of multidrug-resistant (MDR) pathogens [2,3,4,5]. Indeed, up to 76% of patients admitted to PICUs receive antimicrobial therapy, most commonly for perioperative prophylaxis or suspected healthcare-associated infections (HAIs). The most frequently prescribed antibiotics are first-generation cephalosporins (mainly cefazolin), penicillin/beta-lactamase inhibitor combinations, carbapenems, anti-pseudomonal cephalosporins, and glycopeptides [2,3].

Antimicrobial stewardship programs (ASPs) have emerged to optimize antibiotic use, reduce the dissemination of resistance and improve clinical outcomes (including hospital length of stay (LOS) and mortality) [1,2,6]. Effective stewardship requires the identification of the antimicrobial agents most responsible for driving local resistance patterns, followed by the implementation of targeted strategies to reduce their unnecessary use or to guide more judicious prescribing [1]. Evidence-based interventions described in the literature include formulary restriction, prior authorization, antibiotic cycling, facility-specific treatment guidelines with defined treatment duration, and computer-assisted decision-support systems [6,7,8,9]. In adult critical care, multidisciplinary collaboration among intensivists, infectious disease (ID) physicians, and pharmacists has yielded favorable results in ASP implementation, with evidence indicating that stewardship interventions do not increase mortality risk [4,10,11]. Comparable evidence specifically addressing antimicrobial stewardship interventions in PICUs remains scarce. Nevertheless, the broader pediatric hospital ASP literature consistently demonstrates effectiveness in reducing antimicrobial use without adversely affecting inpatient mortality, LOS, or readmission rates, suggesting that similar benefits may also be achievable in the PICU setting [2,5,9,12,13,14].

The few published studies in critically ill pediatric patients describe PICU-ASP teams composed of pediatric ID physicians, pharmacists, microbiologists and pediatric intensivists [2,12]. Prospective audit and feedback, and a handshake stewardship approach, with daily face-to-face rounds, comprehensive prescription review, and collaborative feedback without preauthorization requirements, have demonstrated significant reductions in broad-spectrum antibiotic consumption in the PICU setting, including carbapenems and glycopeptides, without negatively affecting patient safety [15]. Reported outcomes include antimicrobial consumption, measured as days-of-therapy (DOT) and length-of-therapy (LOT) per patient-days; PICU and hospital LOS; total and infection-related mortality; readmission rates within 48 h; and cost of therapy.

Despite the increasing implementation of antimicrobial stewardship programs (ASPs), robust evidence regarding their long-term effectiveness in PICUs remains scarce, and the optimal stewardship model for this highly complex setting has yet to be defined. We therefore evaluated the impact of a multidisciplinary prospective post-prescription review and feedback ASP in a European medical–surgical PICU on antimicrobial use and prescription quality.

## 2. Results

### 2.1. Antimicrobial Use

#### 2.1.1. PICU Activity and Overall Antimicrobial Consumption

During the study period, median monthly activity in the PICU was 475 patient-days (PD) (IQR: 396–534 PD) and the median monthly discharge (D) was 113 patients (IQR: 102–124 patients). These data remained stable, as shown in Supplementary Figure S1. The only notable exception occurred during the COVID-19 pandemic in 2020, when median monthly PD decreased by 24.7% compared with 2019: 361 PD/month in 2020 (IQR: 277–438 PD/month) vs. 480 PD/month in 2019 (IQR: 437–521 PD/month), *p* = 0.003, whereas monthly discharges remained unchanged (2019: 112 D/month (IQR: 98–119) vs. 2020: 112 D/month (IQR: 105–126 D/month, *p* = 0.349)).

The distribution of monthly total antibiotic and antifungal use expressed as DOT/100 PD during the ASP pre-implementation (July 2018–December 2020) and post-implementation periods (January 2020–March 2025) is shown in Supplementary Figure S2.

The following results are derived from the interrupted time-series analysis. To facilitate the interpretation of antimicrobial and antifungal consumption according to the denominator, a supplementary table is provided (Supplementary Table S1).

#### 2.1.2. Antibiotic Use

Despite the reduction in PICU activity during 2020, total systemic antibiotic use showed no significant pandemic-related disruption. There was no pre-pandemic trend (*β* = 0.54 DOT/100 PD/month; *p* = 0.571), no immediate level change at pandemic onset (*β* = +4.57; *p* = 0.776), and no change in the post-pandemic trend (*β* = −0.88; *p* = 0.658). These findings indicate that the pandemic did not substantially alter underlying prescribing patterns, supporting the use of the period between July 2018 and December 2020 as a common pre-intervention baseline for the subsequent evaluation of the stewardship intervention.

**Figure 1:**
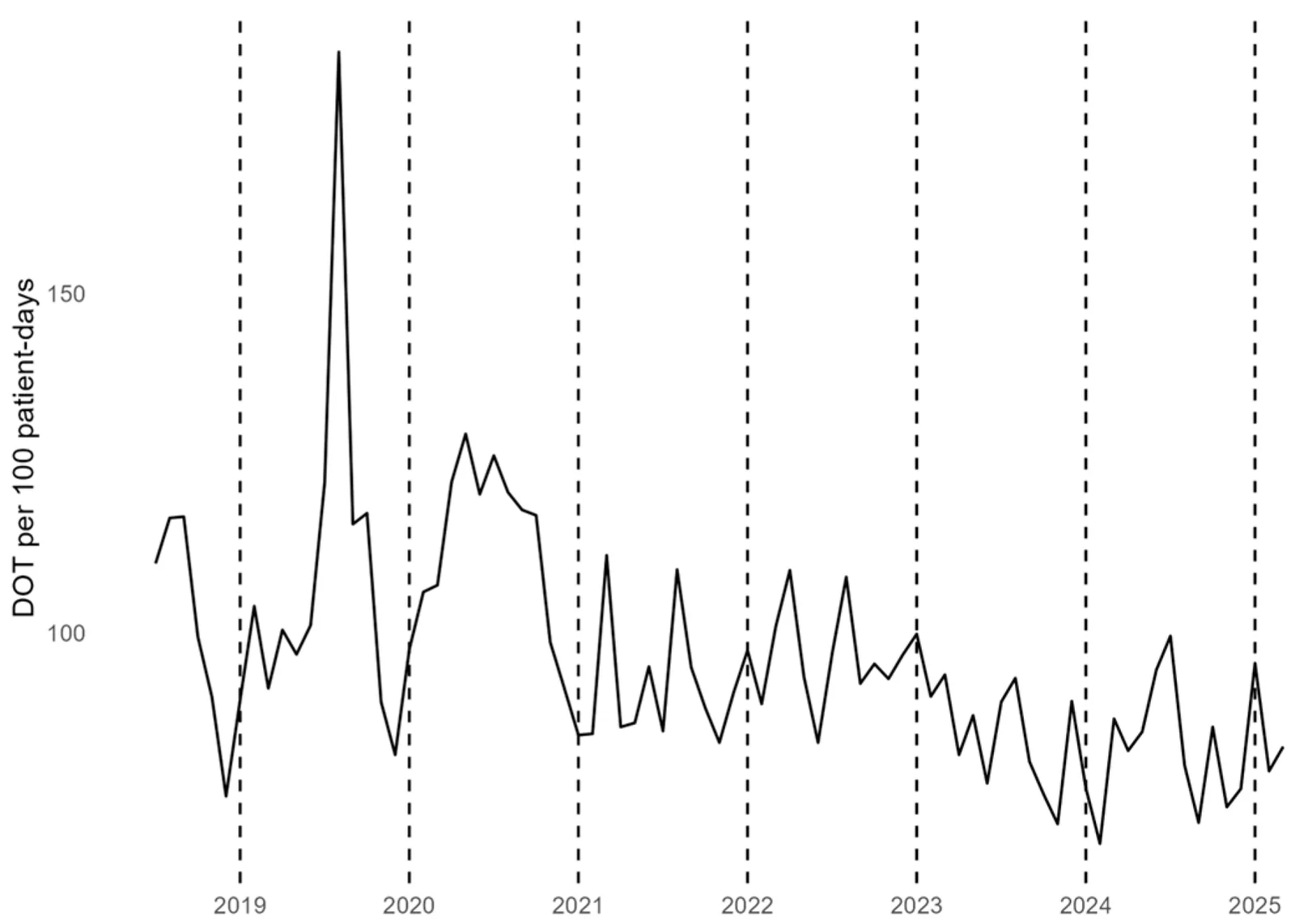
Total antibiotic use over time, expressed as DOT/100 patient-days/month (July 2018–March 2025). Vertical dashed lines mark calendar-year boundaries. The COVID-19 pandemic occurred during 2020. PICU-specific ASP implementation began in January 2021.

Following ASP implementation in the PICU in January 2021, total antibiotic consumption decreased significantly, with an immediate level reduction of −18.2 DOT/100 patient-days/month (*p* = 0.006; 95% CI: −30.7 to −5.7) and a sustained monthly decline of −0.73 DOT/100 PD/month (*p* = 0.028), as illustrated in Figure 1.

When expressed as DOT/100 discharges, the pandemic period was associated with a significant declining trend (*β* = −22.3 DOT/100 D/month; *p* = 0.004), reflecting the reduction in admissions during 2020, while the LOS remained relatively preserved.

After accounting for this pandemic effect, ASP implementation was associated with a positive change in slope (*β* = +20.2 DOT/100 D/month; *p* = 0.002) relative to the pandemic-period slope. The absolute post-ASP slope was −2.1 DOT/100 D/month (−22.3 + 20.2), indicating that antibiotic consumption continued to decrease after ASP implementation, although at a substantially slower rate than during the pandemic period.

**Figure 2:**
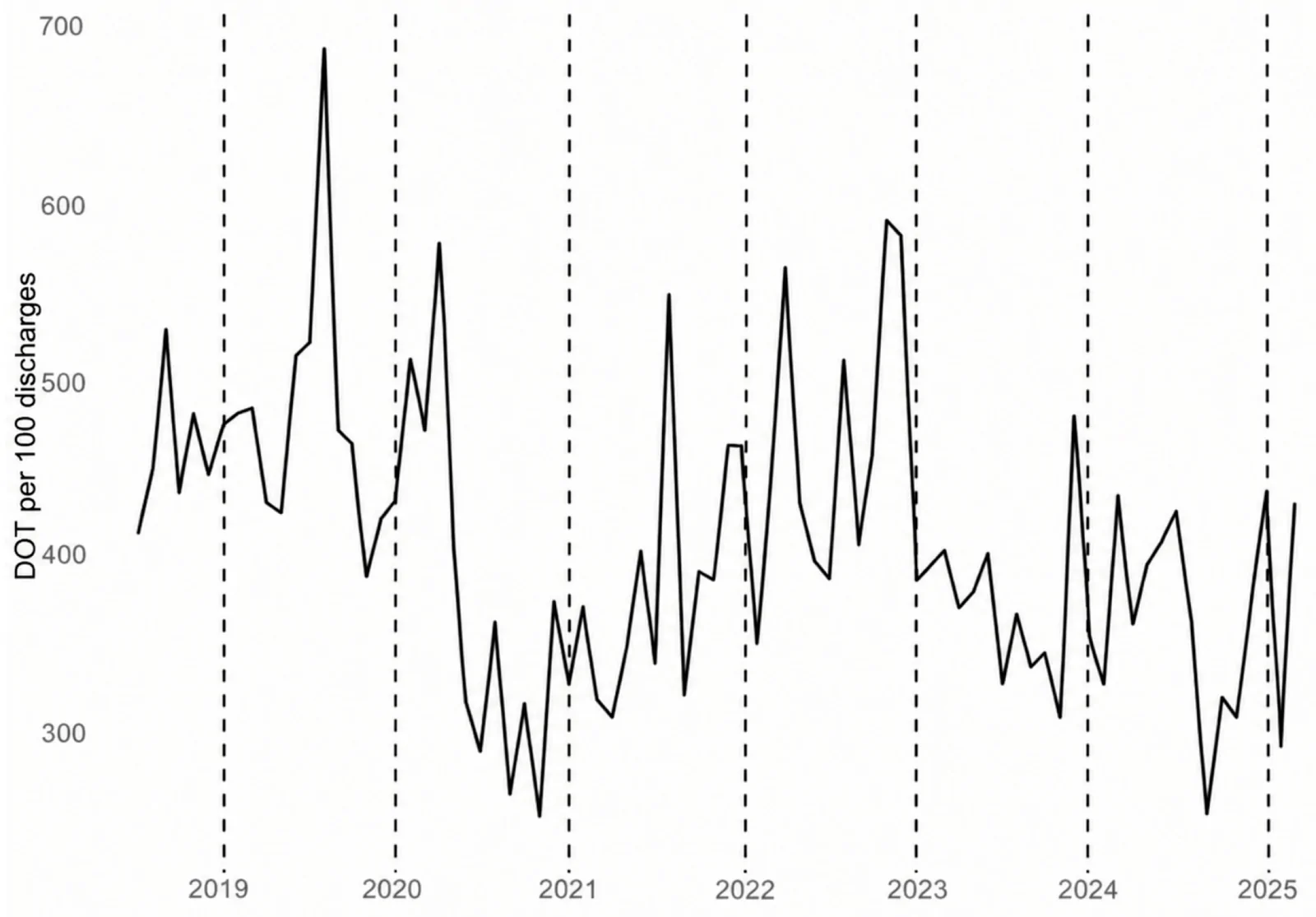
Total antibiotic use over time, expressed as DOT/100 discharges/month (July 2018–March 2025). Vertical dashed lines mark calendar-year boundaries. The COVID-19 pandemic occurred during 2020. PICU-specific ASP implementation began in January 2021.

Furthermore, ASP implementation was associated with an immediate increase in DOT/100 D (*β* = +149.2 DOT/100 D/month; *p* = 0.002). This datum represents a level change relative to the model-based counterfactual trajectory, which was declining during the pandemic, rather than an increase above pre-pandemic prescribing levels. Consistent with this interpretation, post-intervention values remained below pre-pandemic levels, as shown in Figure 2.

**Figure 3:**
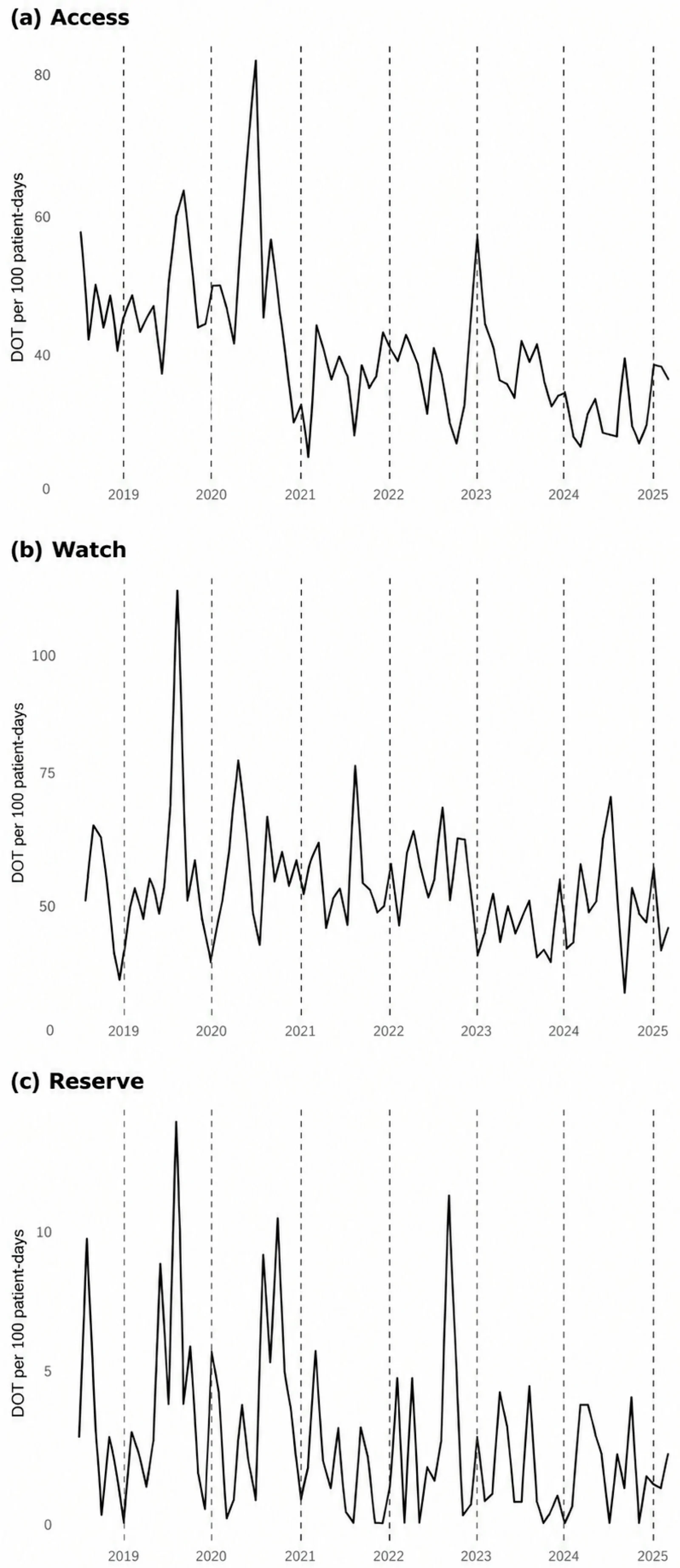
Antibiotic use by WHO AWaRe classification over time, expressed as DOT/100 patient-days per month) (July 2018–March 2025): (**a**) Access, (**b**) Watch, (**c**) Reserve. Vertical dashed lines mark calendar-year boundaries. The COVID-19 pandemic occurred during 2020. PICU-specific ASP implementation began in January 2021.

Antibiotic use by WHO AWaRe category expressed as DOT/100 PD is displayed in Figure 3. *Access* antibiotics showed no significant pandemic-related changes at the immediate level (*p* = 0.371) or in trend (*p* = 0.314). Following ASP implementation, *Access* antibiotic use decreased significantly, with an immediate reduction of −12.8 DOT/100 PD/month (*p* = 0.001; 95% CI: −19.9 to −5.6), without a significant additional change in trend (*p* = 0.173) (Figure 3a). *Watch* antibiotics showed no significant changes related to either the pandemic or the stewardship intervention (Figure 3b). *Reserve* antibiotics were not significantly affected by the pandemic, but following ASP implementation, a small but statistically significant immediate reduction was observed (*β* = −2.50; *p* = 0.047) (Figure 3c).

When expressed as DOT/100 discharges, significant changes were only detected in *Access* antibiotics, including a significant pandemic-related declining trend (*β* = −11.2 DOT/100 D/month; *p* < 0.001), followed by a significant immediate increase after ASP implementation (*β* = +39.1; *p* = 0.027) and a significant positive trend (*β* = +10.8; *p* < 0.001). The absolute post-ASP slope was −0.4 DOT/100 D/month (−11.2 + 10.8), indicating that the decline observed during the pandemic period was largely attenuated after ASP implementation but did not reverse into a sustained increase. The apparent immediate increase should be interpreted as a change from the lower consumption levels observed during the pandemic, rather than as an increase above pre-pandemic levels.

#### 2.1.3. Antifungal Use

**Figure 4:**
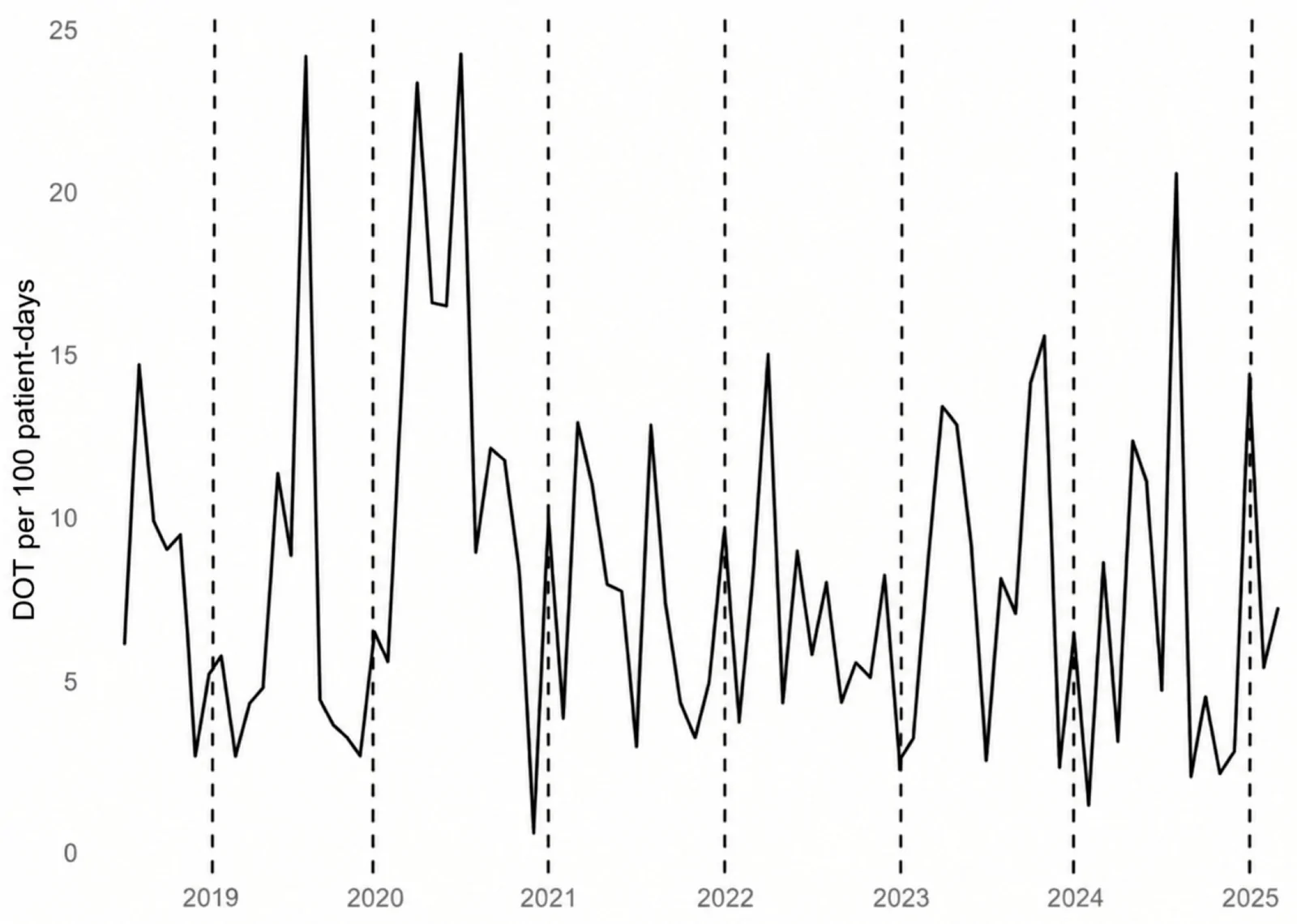
Total antifungal use over time, expressed as DOT/100 patient-days/month (July 2018–March 2025). Vertical dashed lines mark calendar-year boundaries. The COVID-19 pandemic occurred during 2020, and PICU-specific antimicrobial stewardship program (ASP) implementation began in January 2021.

Total antifungal use showed a non-significant increase at pandemic onset when expressed as DOT/100 PD/month (*β* = +9.6; *p* = 0.057) (Figure 4), and pandemic terms were not retained in the subsequent DOT/100 patient-days ASP model. In DOT/100 D/month analyses, the pandemic-associated increase was statistically significant (*β* = +43.1; *p* = 0.016), although the pandemic-related change in slope was not significant (*β* = −2.5; *p* = 0.215). Because of the significant pandemic-related level effect, pandemic terms were retained in the subsequent DOT/100 D ASP model. ASP implementation was not associated with a significant immediate change in antifungal use (*β* = −4.36 DOT/100 PD; *p* = 0.077). A positive post-ASP slope change was observed in DOT/100 D/month (*β* = +3.70; *p* = 0.037). Given the absence of a significant pandemic-related slope change, this positive post-ASP slope-change coefficient should not be interpreted as an increase in antifungal prescribing attributable to the ASP implementation; it reflects a change in the prescribing trend expected based on the previous data. 

#### 2.1.4. Drug-Specific Consumption

In exploratory drug-specific analyses, pandemic-associated increases in DOT/100 patient-days were observed for amoxicillin, azithromycin, clindamycin, micafungin, and voriconazole. After the ASP implementation, significant reductions were observed for selected agents, including amikacin, amoxicillin, azithromycin, cefazolin, and linezolid. Monthly consumption trends (DOT/100 patient-days) for these individual antimicrobial agents are shown in Supplementary Figure S3. Given the large number of individual models fitted, these findings should be interpreted as exploratory.

### 2.2. Prescription Quality

Overall, 616 prescriptions (corresponding to 358 patients admitted to the PICU) were evaluated across the 45 hospital-wide point-prevalence surveys (PPSs) conducted during the study period: 22 before PICU-ASP implementation (326 prescriptions) and 23 post-intervention (290 prescriptions).

Systemic antibiotics accounted for 89.3% of evaluated prescriptions; 6.5% were antifungals and 4.2% antivirals. The parenteral route was the most frequent route of administration (77.3%), whereas 22.0% of the evaluated antimicrobials were administered enterally. The most prescribed agents identified in the PPSs were: amoxicillin–clavulanate (*n* = 86, 13.9%), piperacillin–tazobactam (*n* = 66, 10.7%), cefotaxime (*n* = 57, 9.2%), cefazolin (*n* = 55, 8.9%), and meropenem (*n* = 51, 8.3%).

The 10 most frequent indications for the evaluated antimicrobial prescriptions were prophylaxis (2.7%); clinical/laboratory suspicion of infection (13.1%); sepsis/septic shock (10.6%), community-acquired pneumonia (11.4%); bacteremia of various sources, including central line-associated bloodstream infection (8%); hospital-acquired pneumonia, including ventilator-associated pneumonia (5.8%); meningitis/ventriculitis (4.7%); bronchiolitis (2.6%); suppurative intracranial infections (2.4%); and intraabdominal infections (2.4%).

Comparing all the evaluated prescriptions in both periods, treatment of HAIs rose from 26.7% pre-ASP to 47.2% post-ASP, while treatment of community-acquired infections, surgical prophylaxis, and immunocompromised patients decreased (from 33.7% to 27.9%, from 11.3% to 7.3% and from 6.1% to 4.1%, respectively). The type of treatment (empirical/targeted/prophylaxis) did not change significantly (*p* = 0.43): empirical treatment accounted for approximately half of prescriptions in both periods (49.4% pre-ASP and 51.7% post-ASP); targeted treatment for 24.5% and 27.2% of prescriptions, respectively; and prophylaxis for 25.2% and 21.0% of prescriptions, respectively.

General prescription quality did not improve after ASP implementation, with similar proportions of optimal prescriptions before and after the intervention (89.1% vs. 88.4%; *p* = 0.79). The distribution of ‘optimal’ vs. ‘non-optimal’ prescriptions was similar among prophylactic and therapeutic prescriptions (90.1% vs. 9.8% and 88.4% vs. 11.6%, respectively; *p* = 0.854), both empirical and targeted (88.4% vs. 11.6% in both cases), and both for community-acquired infections and HAI treatment (88.4% vs. 11.6% and 88.7% vs. 11.3%, respectively).

Overall, the most frequent reasons for prescriptions being considered ‘non-optimal’ (*n* = 72) were: inadequate spectrum (47%), not first-choice antimicrobial according to local guidelines (39%), lack of antimicrobial indication (36%), excessive treatment duration (14%), and dosing issues (8%).

When comparing pre-ASP and post-ASP implementation periods, spectrum appropriateness decreased slightly, with adequate-spectrum prescriptions falling from 94.6% to 88.4% (*p* = 0.028), mainly driven by an increase in excessive-spectrum prescriptions (4.8% to 10.1%). Adherence to local guidelines improved (*p* < 0.001), with the proportion of prescriptions for which no applicable local guideline existed decreasing from 15.4% to 4.7% in the post-ASP period. No significant changes were observed in the appropriateness of the antimicrobial indication (94.7% vs. 95.6% indicated; *p* = 0.59), or dosing/interval appropriateness (≥97% adequate in both periods). The planned duration of therapy was not explicitly documented in up to 38% of antimicrobial prescriptions, with no positive change observed after ASP implementation.

### 2.3. Complementary and Balancing Measures

Considering complete calendar years only (2019–2024), mean PICU LOS was 4.6 (standard deviation, SD: 0.5) days and remained stable throughout the study period (range: 4.2–5.4 days), with no evidence of a systematic upward or downward trend. Mean PICU mortality in the same period was 1.4 ± 0.3% (range: 0.92–1.76%), with no statistically significant linear trend over time.

Comparison of antimicrobial resistance between the post-ASP (2021–2024) and the pre-ASP (2018–2020) periods showed a significant increase in the proportion of ESBL-producing *Escherichia coli* (11.2% vs. 18.3%; OR: 1.78, 95% CI: 1.18–2.68; *p* = 0.006) and amoxicillin–clavulanate-resistant *E. coli* (27.2% vs. 36.2%; OR: 1.52, 95% CI: 1.11–2.09; *p* = 0.007). Conversely, the proportion of meropenem-resistant *Pseudomonas aeruginosa* significantly decreased during the post-ASP period (5.9% vs. 2.7%; OR: 0.44, 95% CI: 0.20–0.98; *p* = 0.040). No significant differences were observed in the prevalence of the remaining organisms, as showed in Supplementary Table S2.

## 3. Discussion

In our study, the implementation of a PPRF-based ASP integrated with the hospital-wide ASP in a European medical–surgical PICU was associated with significant and sustained reduction in total antibiotic consumption, without a negative impact on clinical outcomes (LOS, mortality). Taken together, our findings support the growing evidence that multidisciplinary ASPs based on collaborative review can optimize antimicrobial prescribing in critically ill children safely [2,4,9,15,16,17]. Beyond confirming the effectiveness of collaborative stewardship, this study provides long-term evidence from an ITS analysis showing that these benefits were maintained despite evolving patient complexity, associated with the expansion of the PICU and the development of a dedicated pediatric oncology center in the hospital, as well as the unprecedented disruption caused by the COVID-19 pandemic. These results strengthen the evidence supporting stewardship as an integral component of quality improvement in pediatric critical care, rather than solely a strategy to reduce antibiotic consumption.

Although antimicrobial stewardship is now considered a core component of hospital quality improvement, evidence in pediatric intensive care remains scarce. PICUs are among the most challenging settings for stewardship owing to the frequent use of empirical broad-spectrum antibiotics in critically ill children, making them a priority for stewardship interventions. Recent reviews have highlighted the need for PICU-specific evidence, as recommendations from adult intensive care may not be directly applicable to children [3,10,17]. Our study addresses this gap by providing long-term evidence from a medical–surgical PICU using a robust interrupted time-series design.

The reduction in antibiotic consumption following ASP implementation is consistent with previous stewardship studies in pediatric critical care. In our PICU, antibiotic use declined immediately after the intervention and continued to decrease throughout follow-up, indicating sustained changes in prescribing behavior. Similar reductions have been reported after multidisciplinary prospective audit-and-feedback interventions in pediatric [2] and adult ICUs [4], without adversely affecting mortality or length of stay. Likewise, systematic reviews identify prospective audit and feedback as one of the most effective stewardship strategies for reducing unnecessary antimicrobial exposure [9,15,16,17], while long-term interrupted time-series data confirm that these benefits can be sustained over time [18]. Together, these findings support collaborative stewardship as an effective approach to achieving durable improvements in antimicrobial prescribing.

A methodological strength of our study is the simultaneous evaluation of antimicrobial consumption using both DOT/100 PD and DOT/100 D, and the explicit consideration of the COVID-19 pandemic as a potential confounding event. Numerous studies have demonstrated changes in antimicrobial prescribing during the early phases of the pandemic, driven by uncertainty regarding bacterial co-infection rates and secondary infections despite relatively low rates of microbiologically confirmed bacterial disease [19,20]. Our findings show that the metrics DOT/100 PD and DOT/100 D may behave differently during periods of changing healthcare activity, such as the COVID-19 pandemic, when PD declined while admissions remained relatively stable.

PICU activity may also influence antimicrobial prescribing independently of ASP implementation [3]. Changes in admissions, patient volume, or workload may affect prescribing practices and antimicrobial exposure. DOT/100 PD appears to be the more appropriate metric for evaluating stewardship interventions in intensive care, as it better reflects antimicrobial exposure while accounting for fluctuations in patient turnover. The significant reduction observed in our study using this indicator supports a true decrease in antibiotic exposure rather than an artefact of changes in PICU activity.

Analysis of individual antimicrobial agents revealed temporary pandemic-related prescribing changes. Increased consumption of amoxicillin, azithromycin and clindamycin reflected the diagnostic uncertainty that characterized the initial pandemic period, when empirical antibiotic therapy was frequently prescribed for suspected bacterial respiratory co-infections despite the predominantly viral nature of SARS-CoV-2 infection [19,20]. The temporary increase in micafungin and voriconazole use coincided with a transient increase in the admission of pediatric onco-hematological patients transferred from other hospitals to our PICU during the COVID-19 pandemic. Once routine referral pathways were re-established after the pandemic, the case mix returned to baseline and antifungal use rapidly normalized, indicating that this increase reflected a temporary change in patient characteristics rather than a sustained modification of prescribing practices.

An additional perspective of the impact of ASPs is provided by the analysis according to the WHO AWaRe classification. In our cohort, the overall reduction in antibiotic consumption was mainly driven by decreases in *Access* and *Reserve* antibiotics, whereas *Watch* antibiotic use remained stable during the study period [21,22]. Rather than indicating a limited stewardship effect, this likely reflects the appropriate use of these agents in critically ill children with HAIs, previous antimicrobial exposure or severe sepsis. Accordingly, stewardship should focus on optimizing their use through appropriate prescribing, regular reassessment and timely de-escalation rather than indiscriminate reduction. This finding is reassuring, as it indicates that reductions in Access antibiotic use were not achieved at the expense of greater exposure to higher-risk broad-spectrum agents, reflecting a genuine optimization of antimicrobial prescribing.

The significant reduction in *Reserve* antibiotics is particularly relevant, as preserving these last-resort agents is a key objective of antimicrobial stewardship [21]. Because exposure to broad-spectrum antibiotics promotes the emergence of MDR organisms, especially in intensive care, even modest reductions may have important ecological benefits. Our findings therefore suggest that the intervention not only reduced overall antibiotic exposure but also promoted more judicious use of high-impact agents. Analysis of individual antibiotics highlights the selective nature of stewardship interventions. ASP implementation was associated with significant reductions in amikacin, cefazolin, amoxicillin, azithromycin and linezolid, whereas prescribing patterns for other agents remained unchanged, consistent with previous stewardship studies [10,17,18]. The marked reduction in cefazolin likely reflects optimization of surgical prophylaxis, one of the stewardship interventions with the greatest impact on antibiotic consumption [5,17]. Likewise, reductions in amoxicillin, azithromycin and amikacin are consistent with improved management of respiratory infections, earlier de-escalation and greater adherence to local prescribing guidelines.

A key finding of our study is that substantial reductions in antimicrobial exposure were achieved while maintaining prescription quality. Approximately 90% of prescriptions were considered appropriate in both study periods. The baseline level of prescription quality was already high in our PICU, in the context of non-structured ASP support and the involvement of a pediatric intensivist with strong expertise in infectious diseases prior to the formal implementation of the ASP.

The maintenance of a high level of prescription quality indicates that the ASP reduced unnecessary antibiotic use without compromising clinical decision-making, consistent with previous pediatric stewardship studies [5,13,17].

Although the proportion of optimal prescriptions remained stable, adherence to local antimicrobial guidelines improved significantly, while the proportion of non-assessable prescriptions declined. These findings likely reflect the progressive development of institutional guidelines and the continuous educational feedback provided by the multidisciplinary stewardship team, both recognized drivers of sustained improvements in prescribing behavior [6,16,17].

Changes in patient case mix should be considered when interpreting these results. Following ASP implementation, HAIs accounted for a greater proportion of prescriptions, whereas community-acquired infections and surgical prophylaxis became less frequent. This increase in clinical complexity may explain the slight rise in prescriptions considered excessively broad-spectrum, reflecting the greater need for empirical coverage in patients at higher risk of multidrug-resistant pathogens.

Importantly, reductions in antibiotic exposure were not accompanied by adverse changes in clinical outcomes, including PICU length of stay or complexity-adjusted mortality, supporting the growing evidence that prospective audit and feedback safely optimizes antimicrobial prescribing without compromising patient care [4,11,16,17]. These findings reinforce the concept of antimicrobial stewardship as a patient safety intervention rather than solely a strategy to reduce antibiotic consumption [8,16].

Finally, the sustained reduction in antibiotic use together with preserved prescription quality suggests successful integration of the stewardship program into routine PICU practice. Although acceptance of individual recommendations was not formally assessed, the multidisciplinary model, based on close collaboration between intensivists, ID specialists, pharmacists and microbiologists, likely contributed to its effectiveness and sustainability. This observation is consistent with evidence showing that collaborative prospective audit-and-feedback programs achieve greater clinician engagement and long-term success than restrictive preauthorization strategies [10,23,24,25]. The presence of a dedicated PICU antimicrobial stewardship lead likely further enhanced communication among the multidisciplinary team and promoted adherence to local recommendations.

Although antimicrobial resistance is influenced by multiple factors and is difficult to modify over short study periods [16,17], reducing unnecessary exposure to broad-spectrum antibiotics remains a key ecological objective of antimicrobial stewardship. The reduction in *Reserve* antibiotics observed in our study therefore represents an important ecological benefit, although continued surveillance is needed to determine its long-term impact on local resistance patterns.

The finding of an increase in ESBL-producing and amoxicillin–clavulanate-resistant *E. coli* is unlikely to be directly attributable to ASP implementation [5] and may instead reflect the increasing prevalence of resistant Enterobacterales in our setting, including at the community level. These data should be interpreted cautiously, as the impact of ASP interventions on local antimicrobial ecology may take time to become apparent, and causal relationships between ASP implementation and changes in resistance patterns are difficult to establish.

This study has several strengths, including the use of a robust ITS analysis with more than four years of follow-up, explicit adjustment for the COVID-19 pandemic, and a comprehensive evaluation of stewardship effectiveness encompassing antimicrobial consumption, prescription quality, and clinical outcomes. Furthermore, it provides real-world evidence from a large tertiary European medical–surgical PICU, where high-quality pediatric stewardship data remain scarce [3,10,17].

Several limitations should also be recognized. As a single-center study conducted in a tertiary referral hospital, its findings may not be fully generalizable to other PICUs with different patient populations, prescribing practices or stewardship resources. Although ITS analysis is among the strongest quasi-experimental designs for evaluating healthcare interventions, residual confounding cannot be excluded. Prescription quality was assessed using repeated PPSs, which do not capture longitudinal changes within individual treatment episodes. Residual autocorrelation and seasonality were not explicitly modeled and may therefore have influenced the precision of some effect estimates.

Despite these limitations, our findings have important implications for antimicrobial stewardship in pediatric intensive care. Our results suggest that multidisciplinary prospective audit and feedback promotes more deliberate, individualized, and evidence-based antimicrobial prescribing. The combination of reduced antibiotic exposure, preserved prescription quality, and stable clinical outcomes supports the integration of stewardship into routine PICU practice as an essential component of quality improvement and patient safety initiatives. Future multicenter studies using standardized stewardship metrics and patient-level outcomes will be crucial to determine which components of multidisciplinary ASPs provide the greatest benefit in critically ill children and to establish best-practice recommendations for pediatric intensive care.

## 4. Materials and Methods

### 4.1. Study Design

We conducted a prospective quasi-experimental study using interrupted time-series analysis (ITS) to evaluate antimicrobial use trends before (July 2018–December 2020) and after (January 2021–March 2025) the implementation of a multifaceted antimicrobial stewardship program (ASP), integrated within the hospital-wide ASP, in a tertiary PICU in Barcelona, Spain.

### 4.2. Setting

The study was conducted at the Sant Joan de Déu Hospital located in Barcelona, Spain, a tertiary referral children’s hospital with a 24-bed PICU and approximately 1500 annual admissions of patients younger than 18 years of age.

#### 4.2.1. Hospital ASP

The hospital ASP (*Programa de Optimización del uso de Antimicrobianos Sant Joan de Déu, PROA-SJD*) [5] was first implemented in January 2017. The main ASP strategy was prospective prescription review and feedback (PPRF). In addition, the program included educational interventions, such as regular training sessions, dissemination of local antimicrobial prescribing guidelines, and multidisciplinary case discussions. No restrictive antimicrobial measures or mandatory preauthorization policies were applied, but prescription filters for selected antimicrobial agents were incorporated in the hospital e-prescription system, making it necessary for the prescriber to specify the indication.

As part of the ASP, active surveillance of antimicrobial consumption was initiated using days-of-therapy per 100 patient-days (DOT/100 PD) and DOT per 100 discharges (DOT/100 D). In addition, selected microorganisms were monitored as indicators of antimicrobial resistance (AMR), and prescription quality was periodically assessed through point-prevalence surveys (PPSs). Other routinely monitored hospital indicators, including mortality and readmission rates, were also evaluated [6].

The deployment of the program was progressive, initially focusing on surgical and medical inpatient wards and subsequently expanding to the oncology department and the PICU, where ASP intervention started formally in 2021.

The phased implementation of the ASP was partly related to a period of institutional growth and organizational change. In early 2019, during the first months of the study period, the PICU was relocated and expanded, and a new electronic critical care information system was implemented. In 2020, the COVID-19 pandemic also resulted in organizational changes. Subsequently, in 2022, a dedicated pediatric oncology center was developed.

Prior to formal implementation in the PICU, ASP support was provided in a less structured and demand-driven manner, depending on clinical needs and consultation requests from the PICU team.

#### 4.2.2. ASP Intervention in PICU

In 2021, multidisciplinary collaboration was established during rounds 3–4 weekdays per week between pediatric intensivists and a pediatric ID physician from the ASP team, with additional input from the ASP pharmacist and microbiologist. This was a sustained and stable intervention integrated into routine clinical practice since 2021.

The PICU ASP champion, a pediatric intensivist with strong expertise in infectious diseases and a member of the hospital ASP team, was designated as the pediatric intensivist responsible for coordinating antimicrobial stewardship activities within the unit. The champion served as the primary liaison between the PICU and the hospital ASP team, facilitating daily communication, supporting the implementation of stewardship recommendations, and promoting adherence to local antimicrobial guidelines.

All systemic antimicrobial prescriptions were reviewed during the 3–4 weekdays per week dedicated to ASP evaluation. The ASP team provided individualized recommendations aimed at optimizing antimicrobial therapy, following a ‘handshake stewardship’ model. The recommendations were discussed in person (face-to-face) during clinical rounds as part of a shared decision-making process, rather than being presented as external recommendations. The Infectious Diseases physician completed documentation in a standardized form integrated into the electronic clinical chart to indicate whether antimicrobial prescriptions were considered ‘optimal’ or ‘non-optimal’ whenever possible, within the constraints of routine clinical practice and daily workload.

The review focused on the appropriateness of antimicrobial selection, considering patient characteristics, infection type, and drug properties; adequacy of dosing according to age and organ function; the need for therapeutic drug monitoring; appropriateness of microbiological investigations; feasibility of source control; and potential modification of therapy based on clinical evolution and microbiological results, including escalation, de-escalation, early discontinuation, or conversion from intravenous to oral therapy. Acceptance of ASP recommendations was at the prescribers’ discretion.

### 4.3. Outcomes

#### 4.3.1. Antimicrobial Use

Systemic antibiotic and antifungal administration data were extracted from the e-prescription program and were expressed as DOT/100 PD and as DOT/100 D. DOT was totaled for each month and then standardized to 100 PD and to 100 D, using total DP for all admissions in a given month, irrespective of antimicrobial administration. Person time was calculated by subtracting hour and date–time of room exit from hour and date–time of room entry. Duplicated room entries were excluded. An individual patient counted 1-day present on each calendar day.

Analyses included total antimicrobial and antifungal use and antibiotic subgroups based on the WHO AWaRe (Access, Watch, Reserve) classification [22].

#### 4.3.2. Prescription Quality

Prescription quality (PQ) was assessed by means of cross-sectional PPSs conducted in all admitted patients in all units, including the PICU, with any active systemic antimicrobial prescription at 8 a.m. on the day of the survey. The minimum number of PPSs per year was 4. However, additional PPSs were performed in some years according to specific objectives established within the hospital-wide ASP.

The percentages of ‘optimal’ and ‘non-optimal’ prescriptions were evaluated according to local antimicrobial prescribing guidelines and compared based on the intention of the prescription (empirical treatment, targeted treatment or prophylaxis), the reason for treatment (i.e., community acquired infections or nosocomial infections) and reasons for non-optimal prescription (lack of indication for antimicrobial therapy, excessive or insufficient spectrum, non-adherence to local guideline, inadequate dosing, or excessive or insufficient expected and/or actual duration of the antimicrobial treatment).

#### 4.3.3. Complementary and Balancing Measures

Total PICU LOS, complexity-adjusted mortality rates, and AMR rates were included as complementary outcomes.

Antimicrobial resistance rates (AMRs) were obtained from the annual report of the local Microbiology Department and included data on extended-spectrum *β*-lactamase (ESBL)-producing *Escherichia coli* and *Klebsiella pneumoniae*, quinolone-resistant urinary *E. coli*, amoxicillin–clavulanate-resistant *E. coli*, third-generation cephalosporin-resistant *Enterobacter*, carbapenemase-producing enterobacteriaceae, meropenem-resistant *Pseudomonas aeruginosa*, vancomycin-resistant *Enterococcus* and methicillin-resistant *Staphylococcus aureus* isolation rates [6].

### 4.4. Statistical Analysis

Statistical analyses were performed using IBM SPSS Statistics version 32.0 and R software version 4.5.3. Categorical variables were described as counts and percentages and compared using chi-squared or Fisher’s exact tests, as appropriate. Continuous variables were summarized using mean (standard deviation, SD) or median values (interquartile range, IQR), depending on their distribution.

Monthly antimicrobial use, expressed as days of therapy per 100 patient-days (DOT/100 PD) and per 100 discharges (DOT/100 D), was analyzed using interrupted time-series (ITS) segmented regression models. Generalized least squares (GLS) models were fitted using maximum likelihood estimation, including terms for time, the immediate level change, and the change in slope after the interruption of interest. No explicit correlation structure (e.g., AR(1) or ARMA) or seasonal terms were included in the models.

Pandemic-related changes were first assessed using data from July 2018 to December 2020. For each outcome, the subsequent ASP model was specified according to the presence or absence of a statistically significant pandemic-related effect. When a significant pandemic-related effect was identified, the ASP model retained both pandemic and ASP terms, including the immediate level change and change in slope associated with each interruption. The net post-ASP slope was calculated as the sum of the pandemic-related slope change and the subsequent ASP-related slope change. When no significant pandemic-related effect was identified, the full pre-ASP period (July 2018–December 2020) was treated as a single baseline segment and the ASP model included only the pre-ASP trend, immediate ASP level change, and post-ASP slope change.

Analyses were performed for total antibiotic use, total antifungal use, and antibiotic subgroups according to the WHO AWaRe classification (*Access*, *Watch*, and *Reserve*). Additional exploratory models were fitted for individual antimicrobial agents. Regression results are reported as beta coefficients, standard errors, t-values, *p*-values, and 95% confidence intervals for key intervention effects when applicable. All tests were two-sided, and *p* < 0.05 was considered statistically significant. For selected outcomes, pandemic terms were retained despite the absence of statistical significance when prespecified for consistency with the corresponding denominator analysis.

## 5. Conclusions

In this interrupted time-series study, the implementation of a multidisciplinary antimicrobial stewardship program (ASP) based on prospective prescription review and feedback (PPRF) was associated with a significant and sustained reduction in antibiotic exposure in a tertiary PICU, without compromising prescription quality or clinical safety. Importantly, these improvements were maintained over more than four years of follow-up despite changes in patient case-mix and the disruption caused by the COVID-19 pandemic. Together, these findings support the potential for collaborative stewardship models to achieve sustained reductions in antimicrobial exposure in one of the most complex pediatric healthcare settings.

## Data Availability

The original contributions presented in this study are included in the article/Supplementary Materials. Further inquiries can be directed to the corresponding author.

## Supplementary Materials

The following supporting information can be downloaded at https://www.mdpi.com/article/10.3390/antibiotics15090907/s1: Figure S1: PICU monthly activity expressed as patient-days (a) and absolute monthly discharges (b) over the study period (July 2018–March 2025); Figure S2: Distribution of monthly total antibiotic (a) and antifungal (b) use (DOT/100 patient-days) during the pre-implementation period (July 2018–December 2020, including the pandemic) and the post-implementation period (January 2021–March 2025); Table S1: Interrupted time-series analysis of antimicrobial consumption according to denominator; Figure S3: Monthly consumption trends (DOT/100 patient-days) for individual antimicrobial agents that showed significant reductions after the ASP implementation (July 2018–March 2025): (a) Amikacin, (b) Amoxicillin, (c) Azithromycin, (d) Cefazolin, (e) Linezolid. Table S2: Comparison of antimicrobial resistance between the pre-ASP (2018–2020) and post-ASP (2021–2024) periods.

## Abbreviations

The following abbreviations are used in this manuscript:

AMR	Antimicrobial resistance
ASP	Antimicrobial Stewardship Program
AWaRe	Access, Watch, Reserve classification
CI	Confidence interval
DOT	Days of therapy
ESBL	Extended-spectrum β-lactamase
HAI	Healthcare-associated infection
ID	Infectious disease
IQR	Interquartile range
ITS	Interrupted time series
LOS	Length of stay
LOT	Length of therapy
MDR	Multidrug-resistant
MRSA	Methicillin-resistant Staphylococcus aureus
OR	Odds ratio
PD	Patient-days
PICU	Pediatric Intensive Care Unit
PPRF	Prospective prescription review and feedback
PPS	Point-prevalence survey
PQ	Prescription quality
SD	Standard deviation
WHO	World Health Organization
XDR	Extensively drug-resistant
