# Supplementary material for: Impact of a Multifaceted Antimicrobial Stewardship Program on Antimicrobial Use and Prescription Quality in a Pediatric Intensive Care Unit: An Interrupted Time-Series Study
Source: Antibiotics (Basel). 2026 Sep 15;15(9):907. doi: 10.3390/antibiotics15090907 (PMC13603581; doi:10.3390/antibiotics15090907)
Supplement: Supplementary file 1 [file antibiotics-15-00907-s001.zip › antibiotics-4505152-supplementary.pdf]

SUPPLEMENTARY MATERIAL

(a) Patient-days

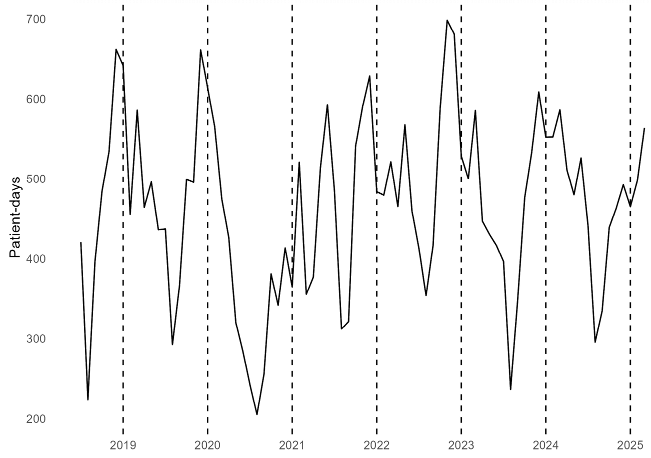

(b) Discharges

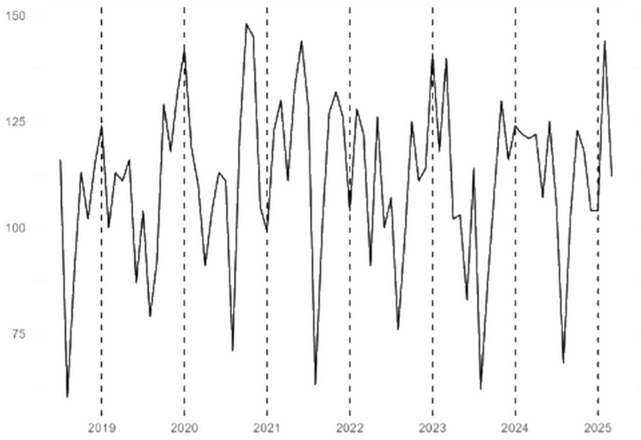

Supplementary Figure S1. PICU monthly activity expressed as patient-days (a) and absolute monthly discharges (b), over the study period (July 2018-March 2025). Vertical dashed lines mark calendar year boundaries. The COVID-19 pandemic occurred during 2020. PICU-specific ASP implementation began in January 2021.

(a) Antibiotics

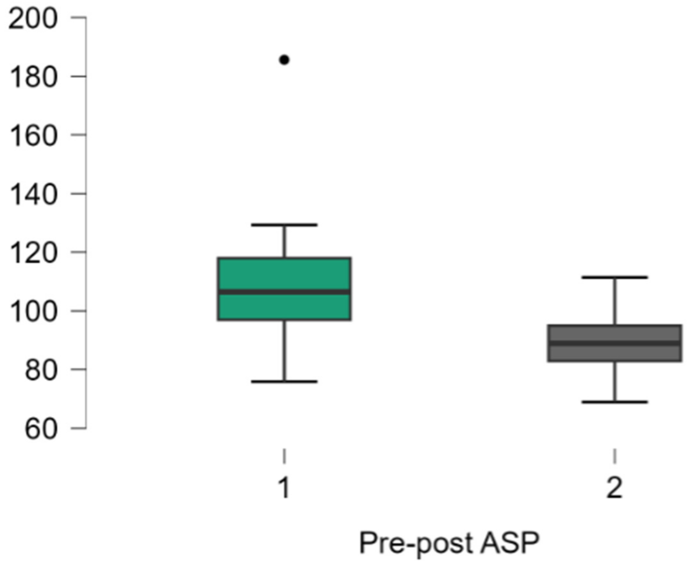

(b) Antifungals

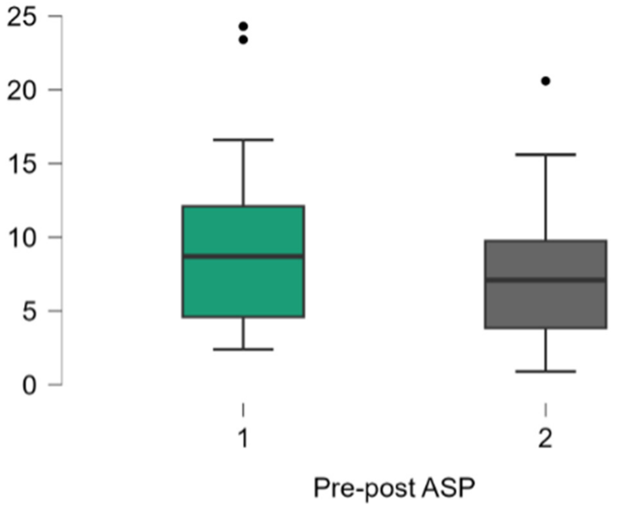

Supplementary Figure S2. Distribution of monthly total antibiotics (a) and antifungals (b) (DOT/100 patient-days) during the pre-implementation period (July 2018–December 2020, including the pandemic) and the post-implementation period (January 2021–March 2025). Boxes represent the interquartile range (IQR), the horizontal line indicates the median, whiskers extend to the most extreme non-outlier values, and points beyond the whiskers represent outliers. The reduction in total antibacterial use across periods is consistent with the significant immediate level change of −18.2 DOT/100 PD ($p = 0.006$) observed in the segmented regression model; the change in antifungal use is consistent with the non-significant immediate reduction of −4.4 DOT/100 PD ($p = 0.077$).

Supplementary Table S1. Interrupted time-series analysis of antimicrobial consumption according to denominator.

Variable	Denominator	Pandemic terms retained in ASP model	Pandemic-related slope change, β/month	ASP immediate level change, β (SE; p)	ASP change in slope, β/month (SE; p)	Net post-ASP slope, β/month
**Total antibacterials**	DOT/100 PD	No	—	−18.2 (6.39; 0.006)	−0.73 (0.32; 0.028)	−0.30
**Total antibacterials**	DOT/100 D	Yes	−22.3	+149.2 (46.12; 0.002)	+20.2 (6.33; 0.002)	−2.1
**Access**	DOT/100 PD	No	—	−12.8 (3.64; 0.001)	No significant change	—
**Access**	DOT/100 D	Yes	−11.2	+39.1 (17.39; 0.027)	+10.8 (2.39; <0.001)	−0.4
**Watch**	DOT/100 PD	No	—	No significant change	No significant change	—
**Watch**	DOT/100 D	Yes*	−10.5	+96.5 (37.28; 0.012)	+9.2 (5.12; 0.077)	−1.3
**Reserve**	DOT/100 PD	No	—	−2.50 (1.24; 0.047)	No significant change	—
**Total antifungals**	DOT/100 PD	No	—	−4.36 (2.43; 0.077)	−0.18 (0.12; 0.139)	−0.005
**Total antifungals**	DOT/100 D	Yes	Not reported†	Not significant	+3.70 (1.74; 0.037)	—

ASP: antimicrobial stewardship program; D: discharges; DOT: days of therapy; PD: patient-days; SE: standard error.

*For *Watch* antibiotics expressed as DOT/100 D, pandemic terms were retained in the final model despite the pandemic-related slope change not reaching statistical significance, consistent with the original modelling strategy.

†For total antifungal use expressed as DOT/100 D, the manuscript reports a statistically significant pandemic-associated increase of β = +43.1 DOT/100 D, p = 0.016. This coefficient is a pandemic-related level change, not a slope change, and is therefore not placed in the pandemic-slope column.

(a) Amikacin

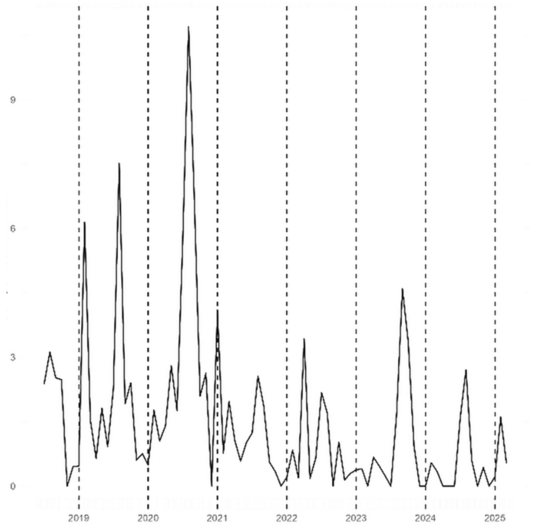

(b) Amoxicillin

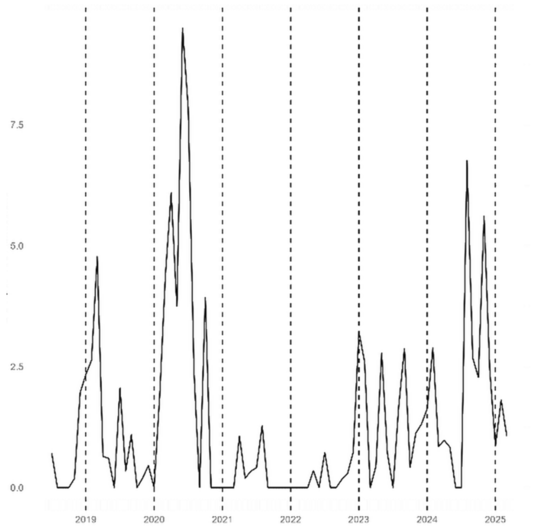

(c) Azithromycin

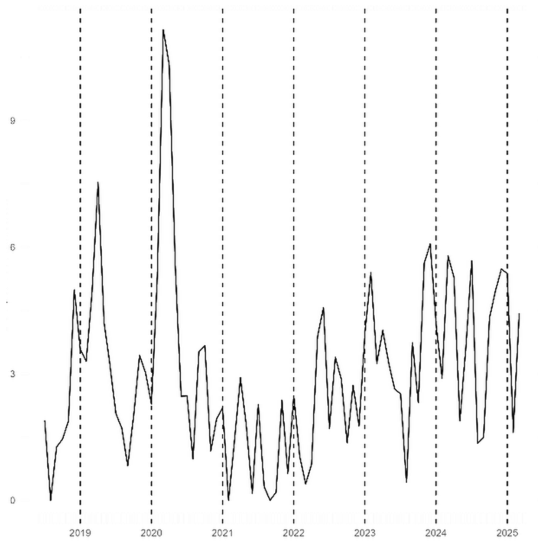

(d) Cefazolin

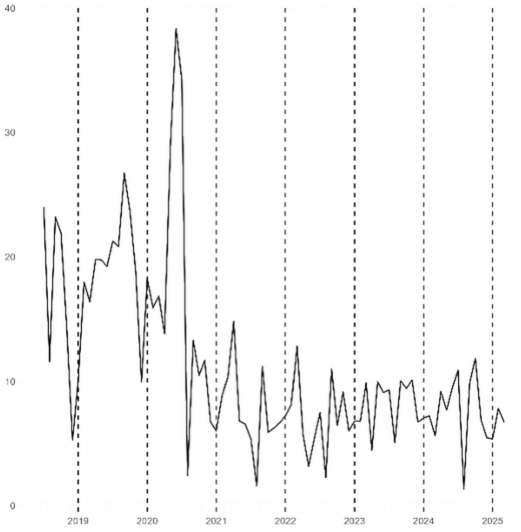

(e) Linezolid

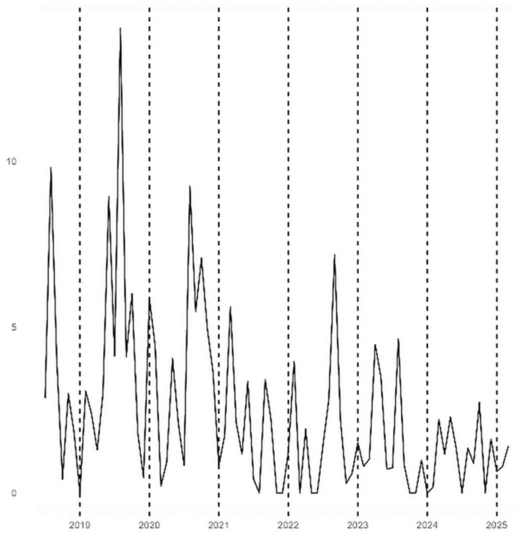

Supplementary Figure S3. Monthly consumption trends (DOT/100 patient-days) for individual antimicrobial agents that showed significant reductions after the ASP implementation (July 2018–March 2025): (a) Amikacin, (b) Amoxicillin, (c) Azithromycin, (d) Cefazolin, (e) Linezolid. Vertical dashed lines mark calendar year boundaries. The COVID-19 pandemic occurred during 2020. PICU-specific ASP implementation began in January 2021.

Supplementary Table S2. Comparison of antimicrobial resistance between the pre-ASP (2018–2020) and post-ASP (2021–2024) periods. Data are presented as n/N (%). Odds ratios compare post-ASP (2021–2024) versus pre-ASP (2018–2020).

**Microorganism**	**2018–2020 n/N (%)**	**2021–2024 n/N (%)**	**OR (95% CI)**	***p* value**
Escherichia coli ESBL	37/331 (11.2)	92/503 (18.3)	1.78 (1.18–2.68)	**0.006**
Klebsiella pneumoniae ESBL	30/214 (14.0)	74/402 (18.4)	1.38 (0.87–2.19)	0.166
Escherichia coli Amoxicillin/clavulanate-resistant	90/331 (27.2)	182/503 (36.2)	0.96 (0.59–1.57)	**0.007**
Enterobacter spp. 3GC-resistant (AmpC)	41/171 (24.0)	43/185 (23.2)	1.72 (0.72–4.12)	0.871
Enterobacterales Carbapenem-resistant	7/1003 (0.7)	19/1587 (1.2)	0.43 (0.19–0.98)	0.214
Pseudomonas aeruginosa Meropenem-resistant	15/253 (5.9)	10/375 (2.7)	1.62 (0.81–3.25)	**0.040**
Pseudomonas aeruginosa MDR	12/253 (4.7)	28/375 (7.5)	2.03 (0.08–50.05)	0.170
Pseudomonas aeruginosa XDR	0/253 (0.0)	1/375 (0.3)	2.33 (0.54–10.16)	1.000
Acinetobacter baumannii Multidrug-resistant	3/26 (11.5)	7/30 (23.3)	3.60 (0.78–16.61)	0.310
Enterococcus faecium Vancomycin-resistant	2/184 (1.1)	10/263 (3.8)	1.00 (0.71–1.43)	0.134
Staphylococcus aureus MRSA	59/455 (13.0)	89/684 (13.0)	1.78 (1.18–2.68)	0.983

ESBL, extended-spectrum β-lactamase; MDR, multidrug-resistant; XDR, extensively drug-resistant; MRSA, methicillin-resistant *Staphylococcus aureus*.
